# Intersectional perspective in elderly care

**DOI:** 10.3402/qhw.v11.30544

**Published:** 2016-05-09

**Authors:** Marta Cuesta, Margareta Rämgård

**Affiliations:** 1 Sociology, Academy of Health and Welfare, University of Halmstad, Halmstad, Sweden; 2 Department of Care, Malmö University, Malmö, Sweden

**Keywords:** Power, intersectionality, focus group, elderly care, ethnicity, gender

## Abstract

Earlier research has shown that power relationships at workplaces are constructed by power structures. Processes related to power always influence the working conditions for (in this study in elderly care) the working groups involved. Power structures are central for intersectional analysis, in the sense that the intersectional perspective highlights aspects such as gender and ethnicity (subjective dimensions) and interrelates them to processes of power (objective dimension). This qualitative study *aims* to explore in what way an intersectional perspective could contribute to increased knowledge of power structures in a nursing home where the employees were mostly immigrants from different countries. By using reflexive dialogues related to an intersectional perspective, new knowledge which contributes to the employees’ well-being could develop. Narrative analysis was the *method* used to conduct this study. Through a multi-stage focus group on six occasions over 6 months, the staff were engaged in intersectional and critical reflections about power relationship with the researchers, by identifying patterns in their professional activities that could be connected to their subjectivities (gender, ethnicity, etc.). The *result* of this study presents three themes that express the staff's experiences and connect these experiences to structural discrimination. 1) Intersectionality, knowledge, and experiences of professionalism; 2) Intersectionality, knowledge, and experiences of collaboration; and 3) Intersectionality, knowledge, and experiences of discrimination. The result demonstrates that an intersectional perspective reinforces the involved abilities, during the conversations, into being clear about, for example, their experiences of discrimination, and consequently developing a better understanding of their professionalism and collaboration. Such deeper reflections became possible through a process of consciousness raising, strengthening the employee's self-confidence, in a positive way.

Globalization of economic structures has contributed to increased reductions in the public sector, which has brought into existence a widespread market for private actors in the field of elderly care. In Sweden, this takes place through private healthcare companies being contracted by primary municipalities in the public sector. The increased global exchange of goods and services has also brought about international migration, affecting the care of elderly individuals (Fagerberg & Engström, 2012; Forsell & Torres, 2012; Svanström, Sunder Johansson, Berglund, & Westin, 2013). One such example is social housing where older immigrants are looked after by staff from different ethnic groups, using ethnicity as a commodity for marketing.

Previous research shows that personal and professional values are often taken for granted by different professional groups in elderly care (Rämgård, 2010; Rämgård, Blomqvist, & Petersson, 2015). These have often been related to how relationships within the healthcare sector have developed into power relationships between staff and recipients of care (Daly & Szebehely, 2011). Some research on gender has also shown how this taking for granted is influenced by differentiating aspects of ethnicity and gender in healthcare (Lill, 2007). Research has also shown that these power relationships are connected to structural processes in the working group, and with the conditions for those working with older people (Eliasson-Lappalainen, 2011). Few studies have described how gender and ethnicity are reproduced in professional groups in elderly care in a more complex context (Torres, 2008). No study has been found that involves an intersectional perspective in the research, particularly by focusing on the employees’ well-being.

Earlier research on intersectionality shows that the “conduct” or “actions” of an individual can depend on “personal experiences” on multiple levels, as well as on different social circumstances in society (Acker, 2008; Eriksson-Zetterquist & Styrhe, 2007). Intersectionality concerns how these different power structures mutually construct each other (Cuesta, 2011). Intersectionality can therefore be understood as an “interactive approach,” as it frames structural “differences” from different “subjective perspectives.”An intersectional approach (which in this study integrates a perspective within the methods connected to data collection as well as the analysis of the material) is thus an attempt at capturing multiple identities in a group of individuals and relating these to power relationships (Crenshaw, 2003). Conversations and meetings between people include different dimensions of gender, ethnicity, sexuality, nationality, and age (De los Reyes & Mulinari, 2005; Likke, 2007, 2010). Researchers can, through an intersectional perspective, contribute to critical dialogues by integrating theory and practice with the aim of increasing our understanding of these different dimensions. Through an intersectional perspective, fragmented perceptions are made visible and turn into more contextualized discourses. Research on “power and inequality” is going through important qualitative changes. A great deal of knowledge production is rooted in social orders, which run the risk of being interpreted in relation to legitimating or discriminating ideas when these are not made visible by research (De los Reyes & Mulinari, 2005).

Through critical discussions in the form of consciousness-raising intersectional conversations between research and practice, these processes can be changed and made visible. Engaging an intersectional perspective would contribute to an in-depth understanding and development of new knowledge about subjectivity (gender, ethnicity, etc.).

## Aim

The aim of this article is to contribute to staff well-being in workplaces by asking in what way an intersectional perspective can contribute to increased knowledge of power structures? The study focuses on the staff's reflections about their experiences, connected, for example, to their subordinate positions at their work place in elderly care.

## Method

Qualitative methodological processes contribute to an understanding, which is based on both proximity and distance, in the sense that knowledge is produced from different perspectives, but around a specific focus area. Using focus groups is an appropriate method to investigate issues where information is processed from a specific level to reflexive contexts of in-depth knowledge and generalizations. But, without recognizing and reflecting issues of subjectivity (gender, ethnicity, etc.), talking to professionals in elderly care, there could be a risk that the dialogues only confirm pre-existing processes of the current inequality, in the care organization, as well as in the society.

The research, by means of a qualitative pilot study, was conducted in 2012. The pilot study was designed in various phases, 1) defining the problem area, 2) planning the role of the researchers in the collaboration, 3) documenting the conversations in the focus group, and 4) analysing the material.

The study was carried out as a multi-stage focus group, in which the same group reflects about specific problem areas over several meetings (Hummelvoll, 2010). The focus group met on six occasions over 6 months, with each session lasting 4 h. The focus groups were conducted through unstructured dialogues (Wibeck, Öberg, & Abrandt-Ahlgren, 2007). The starting point of the conversations in the focus group concentrated on the staff experiences of their professional life in the care unit.

**Figure 1:**
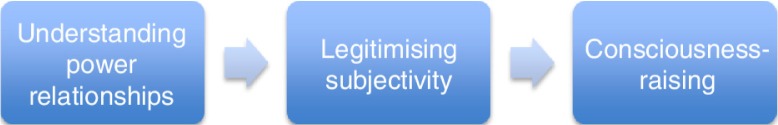
Intersectional focus group.

The focus group was run with an intersectional perspective (see Figure 1). The main purpose was to listen to members and get a free dialogue in which the researcher's role was to lift the intersectional perspective in the dialogue. The aim of the intersectional conversations in the focus group was to reach critical reflections about power relationships by identifying patterns in professional activities that could be connected to subjectivity (gender, ethnicity, etc.). These reflections should then contribute to a social dynamic in the focus group in terms of “consciousness raising,” by developing a deeper understanding on the concrete work situations and connecting them to structural discrimination.

The staff contribute with descriptions of their work, and considers their position in relation to one another, in order to achieve more in-depth conversations about power relationships at work. Questions related to the experiences of the employees were then discussed through illustrations and reflections about care, and subjectivity (gender, ethnicity, etc.), by the researchers. Through these intersectional conversations, the staff become more conscious about their roles and about their professionalism.

The pre-understanding of the researchers is of great importance and contributes to understanding the complexity of the conversations. This pre-understanding is central in relation to the qualitative processes as it, among other effects, reinforces the dynamic of the group. The previous knowledge of the research group influences their different roles and actions, partly through differences based on experiences but also in that they adopt different positions in relation to the narratives.

### Participants

The participants in the focus group were nurses (2) and assistant nurses (8) (one of whom was a man), who were all working at different wards at this particular care institution. Only three individuals in the focus group were born in Sweden, with the remainder coming from different countries in southern Europe, Middle East and North Africa. The participants were aged between 25 and 55 years.

### Data collection

Through the R&D unit of Scania's Association of Local Authorities, the researchers established contact with a privately owned nursing home for elderly immigrants with dementia. The company managing the care institution was given written and oral information regarding the study. The researchers informed the management at an early stage that the participants in the focus group should be of different genders and ethnicities, as well as coming from different wards of the home. The head nurse then consulted the staff verbally and in writing.

### Data and analysis

Data from focus groups have been collected by a narrative analysis (Hyden & Hyden, 1997). By choosing a qualitative method, the researchers are situated close to the informants’ narratives throughout the analysis. The analysis of the narrative has been inspired by Wibeck et al. (2007) and Wilkinson (1998). The material has been transcribed and read several times by the researchers. First, stories between the staff and the researchers were identified in the material to see context and get an understanding of its meaning. These stories were then analysed to see to arguments and what values these arguments are based on and how these are expressed by the participants related to an intersectional perspective. Further, the analyses were conducted to a more abstract level, looking at the conflicts expressed through the stories and put them into themes (Wibeck et al., 2007). These themes can be described as the central focus points of the conversations that simultaneously reflect the participants’ process of understanding. The three themes that emerged relate to descriptions of different work situations and illustrations of different experiences. Due to the researchers having different pre-existing knowledge and representing different fields of research, the presentation of the analysis also reflects the researchers’ different positions in the focus group. The three themes are illustrated by narratives instead of traditional quotes, to highlight the dialogues between the researcher (Researcher 1, Researcher 2) and the staff (Person1-2-3-4-5-6-7-8-9-10).

The narratives illustrate how the intersectional conversations reinforce knowledge about the power situation. It is important to highlight that the employees' reflections are presented by their own voices with the ambition to follow an intersectional perspective point of departure, which has to be understood as a process about the construction of knowledge of the subjectivity, particularly, in this study, about power relationships. After illustrations of the narratives follows an interpretation by the researchers in each theme.

### Ethical aspects

The study was approved by the Ethical Committee (2012:72). The ethical consideration of Helsinki was followed. For this study, all participants were individually informed, verbally and in writing, of the purpose and methods of the study. Their participation was voluntary, and they could withdraw at any point. All materials have been decoded and made anonymous before publication. The materials have been stored under lock and key by the researcher. The materials were shown to the focus group participants before the management was informed of the results.

## Findings

The selected narratives from the analyses resulted in three themes (through selected conversations): *Intersectionality, knowledge, and experiences of professionalism*, concerns the socialization processes connected to the professional activity of the staff; *Intersectionality, knowledge, and experiences of collaboration*, concerns professional activity in relation to staff collaboration; *Intersectionality, knowledge, and experiences of discrimination*, concerns discrimination in relation to the professional activity of the staff. After the narratives follows an explanation that focuses on the interactions between the staff and the researchers.

### Intersectionality, knowledge, and experiences of professionalism

The conversation below illustrates the staff feelings and experiences about power relationships connected to their roles in their workplace. The conversation also illustrates how the intersectional conversations lead to critical reflections and deeper understanding.

(Researcher1) Tell us about how you work?

(Person1) Trust is the most important thing in our work. Between colleagues, it contributes to assignments not being seen as competitions.

(Researcher2) Do you mean that professionalism can be connected to a work ethic and that is what's rewarded?

(Person2) Yes, the fact those assignments become part of a learning process that everyone contributes to, and this leads to a sense of pride.

(Person3) But this process is also characterised by stress (…) you know, everything concerning the constant reorganisations (the roles of short-term employees) etc.

(Researcher2) I just have to understand this! You for example, you were a short-term employee and then became a permanent employee?

(Person4) Yes, it feels right to work here (…) I started working here on a temporary basis, and then it turned into permanent employment, as for most of us.

(Person5) I feel powerless (…), you feel powerless in relation to the demands of life.

(Researcher1) Do you feel powerless from a purely professional standpoint?

(Person6) There is so much pain and anxiety (regarding death); it takes so much time to argue with the hospital, since they often do not want to receive our patients, since they are close to death.

(Researcher2) Is this a general problem in connection with end-of-life care?

(Person6) I feel like there is no awareness of the human being behind everything that is happening.

(Person3) I feel powerless and I become sad from being frustrated about situations, about ‘feeling inadequate’.

(Researcher2) If I understand you correctly, there are many situations that affect your professional activities. (…) You are harboring a lot of frustration.

(Person1) Yes, we are.

(Person8) I think a lot about why we don't show our emotional weak sides at work.

(Person9) We don't have time for reflection.

(Researcher1) Maybe what we are talking about here can help you realise how you carry out your work!

The conversation above entails social interactions between researcher and staff. In the quotations above, (R1) starts the conversation by asking the staff members to describe their work. (The staff) define trust placed in the working group as something essential, which (R2) interprets as them presenting a work ethic. (The staff) reflect on this work ethic originating in an experience-based learning process. They also describe how they are socialized in the course of their professional activities by their peers, even when their work is characterized by stress related to constant reorganizations and staff changes. When (R1) asks how they were recruited to start working at the home, it emerges that all of the employees with foreign backgrounds started as short-term employees before being offered permanent employment. That means they are forced to adapt to the prevalent norms and values of the existing organization in order to receive permanent employment. (One member of the staff) talks about often feeling powerless in relation to life and death in their work. (R1) asks if this is due to feeling powerless purely professionally, since she has heard this in other conversations with staff in elderly care. (All the staff) agree that their feelings of powerlessness are due to a lack of care resources in society. (R2) demonstrates that she does not possess this knowledge herself, and by asking them if they see this as a general problem in connection with end-of-life care. (The staff) express irritation about inequality in society, to exemplify this one of the nurses said, “the human being” is not valued in society, and (R2) interprets these feelings as something that affects their professional activities and makes them frustrated. (The staff) reflect then on not feeling comfortable expressing their emotions and on not being given any time for reflection in the course of their work. (R1) concludes that the reflections in the focus group can create an improved understanding of how they carry out their work.

### Intersectionality, knowledge and experiences of collaboration

The following conversation illustrates the staff feelings and experiences about power relationships connected to collaboration in their working group. The conversation also illustrates how the intersectional conversations lead to critical reflections and deeper understanding.

(Person9) A colleague that I work with – she does things a bit differently. I don't have time to do everything when she is with me. This stressed me! Now I do things differently, I set limits. Once I made this decision I felt like I grew as a person.

(Researcher2) The commitment regarding different tasks looks different, and this affects your collaboration indirectly, and you make the interpretation that different ways of working in a group can lead to an unfair distribution of work.

(Person3) The problem becomes worse when a short-term employee comes along, I was working with my regular colleague this weekend, and everything went off without a hitch.

(Researcher1) I hear you talking in a way that places guilt on you as a group.

People working in healthcare have ‘soft hearts’, we want the best for our elderly.

(Researcher2) But, if we talk about the workplace, how do you process these feelings of guilt?

(Person5) There are intense things that can happen, that cause feelings of vulnerability.

(Person7) Yes, as a coordinator, I know why my colleagues sometimes change wards. Things can happen (…) we are only human. For example, when a colleague is involved in a difficult situation and word gets around (…). Most of the people here have similar experiences.

(Researcher2) We women sometimes complicate things and put our emotions too much into our work. But there are feelings of guilt in relation to all situations, because that's present in all relationships.

(Researcher1) I think that caring requires a special way of working ‘that is ethical’. ‘Taking care of someone else’, that is a special kind of ethic in healthcare work.

(Person10) Do you mean that it is inherent in our roles?

(Person5) Now I begin to think, I want to talk about these things.

The conversation above entails social interactions between researcher and staff. The staff reflect on not being able to manage their work in time through the routines established by the employer. The staff visualize discontent and exchange ideas and compare each other's efforts, and the solution is to create limits with regard to each other in order for the distribution of work to become more uniform and fair. (R2) facilitates the dialogue asking about their commitment to their work in a working group being different, and the conversation turns to the subject of it rather being the difficulty in maintaining continuity in the staff that causes the problem. (R1) reflects that she often hears the group members blame themselves for not being able to manage the care activities expected of them. (One member of the staff) responds that this has to do with their personality, since those who work in care want to provide good care. (R2) raises the question of whether or not this is a workplace where this issue of varying efforts should be discussed in the organization, to which the group says that they do not want to expose each other to emotional pressure. (R2) validates the group by pointing out that feelings of guilt are always present in relationships, and (R1) reflects that there may be a special ethic between healthcare staff. The group then stops positioning themselves as inadequate and instead starts to pose questions to the researchers about their professional roles and the meaning of those.

### Intersectionality, knowledge, and experiences of discrimination

The following conversation illustrates the staff feelings and experiences about power relationships connected to their subjectivity (gender, ethnicity, etc.). The conversation also illustrates how the intersectional conversations lead to critical reflections and deeper understanding

(Researcher1) Here and now, we are going to talk about what happens when you work together. How you share responsibility and how you collaborate? How do you perceive that, at first, management invited only employees with different ethnic backgrounds?

(Person8) I feel annoyed, because I thought we were going to be a mixed group.

(Person1) Equality should be the rule in the workplace and in research.

(Researcher2) It is important for us to know how you perceived this, in the sense of being able to correct situations afterward.

(Person5) We could turn around and go back and demand to start over, but now we did change the group so that more Swedes were included.

(Person10) If we are not supposed to mix, why am I here? I'm a man, but I also feel singled out.

(Researcher1) What we are discussing here definitely has to do with concrete examples of discrimination in professional life. Some decisions depend on the management, and others depend on how you interpret them.

(Researcher2) How can it be, that you as a man took a job in elderly care?

(Person8) As a man, you immediately get a job at the hospital; all the men who have worked here have gone straight on to the hospital.

(Researcher1) Why is that?

(Person8) I think that is has to do with urgent situations not happening here, like they do at the hospital. Around here, it's all about household chores, so to speak (laundry, dishes, cooking and cleaning).

(Researcher1) What do you think of mixing genders in the workplace?

(Person1) Good! Male and female. We are different. As women will often get stuck on our problems.

(Person4) Men are more direct. When there are only women around there is bickering, a lot of bickering about small things.

(Researcher2) Wait a minute, as a gender studies specialist I can say that when women bicker, it is because something is happening, such as discontent.

(Researcher1) Can we now talk about any examples connected to ethnicity?

(Person7) It is advantageous to have different cultures and that employees have different ethnic backgrounds. As a Swede and a Christian, I have no knowledge about Muslims, for example, I know nothing about Ramadan. It is good to be able to come closer to different rituals from different cultures. It is impossible for us to know everything. It is a good opportunity for us to complement each other.

(Person9) For me it is not a problem. I don't say like, here's a Turk, here's a Yugoslavian. When I am at work I give one hundred percent, that is, I'm here more than I am at home. Everyone is my colleague and we spend time together.

(Person6) I have heard of a dark-skinned nurse being treated in a racist way. That was residents.

(…) but relatives can also turn to Swedes (…)

(Person1) I think it is all right, for example, that not everyone knows that it is traditionally cinnamon roll day on Thursdays!

(Researcher1) I think it is interesting and exciting to see how you contribute to making this society (…) more “grown-up.”

The conversation above entails social interactions between researcher and staff. The staff reflect and (R1) starts by pointing out that the reflections in the group are to be a part of their everyday activities and asks how they perceive the focus group based on having another ethnic background than Swedish. (The staff with a non-Swedish background) express considerable irritation with not being a more mixed group, and with being judged based on their ethnicity. Most of all, they feel that it is a form of exclusion when their manager states that at work immigrant staff have a specific need to talk about their work. (R2) then wonders if this is something that can be corrected through a dialogue. (One of the staff) mentions that the society is supposed to be integrated and wonders why research discriminates by looking at specific groups. They do not perceive their problems in the workplace as being related to their ethnic background. (R1) comments that certain decisions in this regard also rest with the management but that it also depends on how the group interprets the decisions of the management. She relates this to workplaces having mostly female employees. (Most of the staff) respond that this is because men are prioritized at the hospital, due to the higher status of the specialized emergency medical care. They explain that is depending on the fact that women and men function differently, such as women not focusing on concrete solutions, but instead turning over problems in their minds again and again. (R2) wonders if this might be the result of structural subordination, and asks the staff with a non-Swedish background if they experience that their different ethnic backgrounds can affect collaboration. (The staff with a Swedish background) reply that it can certainly be good to know a bit about rituals concerning holidays, deaths, etc., but those with a non-Swedish background feel that ethnicity does not create any relationship problems in the workplace and that racism is present in the rest of society, and in relatives of the patients, but not between members of the working group. (R1) then points out that their interpretations and attitudes represent a good example for the rest of the society.

## Discussion

The three themes presented in this study were extracted from the data with the ambition to illustrate how a process of knowledge construction related to the staff experiences not only contributes to a better understanding of their professionalism and collaboration but also about their experiences of discrimination.

The intersectional perspective contributed to a consciousness-raising process developing a better understanding connected to power structures. Talking about power processes, the staff argue that they always do their work properly despite the dearth in their resources, but after more developed reflections connected to the focus group, they also come to the conclusion that their feelings of powerlessness arise from a lack of care resources in society. During the focus group, the staff continued discussing that the routines established by the employer did not give them enough time to manage their collaborative work. But, after more developed reflections upon this item, the group started positioning themselves, asking questions to the researchers about their professional roles and the meaning of those.

During the focus group, the staff also discussed their experiences of discrimination. They, for example, first argued that they did not perceive their problems in the workplace as being related to their ethnic background, but after more developed reflections they concluded that they are still identifying structural subordination related to their gender and ethnicity. Even the focus group did not solve all their problems, the developed reflections during the focus groups made them feel more comfortable and strengthened them to achieve a better collaboration, contributing to their well-being as a group.

The discussion below concentrates on the contribution of the intersectional perspective as a consciousness-raising process. In other words, it analyses how intersectional conversations, in this study, contributes to the employees’ better understanding of how their professionalism and collaboration is constructed by power structures.

Contextualizing the understanding of power relationships and giving it legitimacy by (re)telling narratives about (in)equality requires a critical insight into how the needs of the power structure can be linked to different dimensions of subjectivity (gender, ethnicity, etc.), and how these are integrated at different levels of society (in this case to a workplace) (Cuesta, 2011; Eriksson-Zetterquist & Styrhe, 2007). This study shows that this presupposes an intersectional perspective that focuses on gender, ethnicity, and power relations as an illustration of how subjectivity (in a workplace) reflects structural dimensions, which affect the involved well-being.

The researchers contribute to this understanding by reflecting over the staff's views and asking them questions and legitimating their thoughts. The staff contributed to the focus group in a responsible manner through an honest and self-critical attitude, which shows that they are strong and responsible women who take responsibility for their work. During these sessions, the entire group's awareness of how a collective (in this case tied to a workplace) integrates subjectivity (gender, ethnicity, etc.) into its social structure, and how these different dimensions affect each other, increased by using an intersectional conversation between the staff and the researchers. Through an intersectional perspective that focuses on gender, ethnicity, and power relations, the staff could critically reflect on how structural power relationships affect their shared professional activities, and collaboration in their daily working life. This understanding also brings legitimacy to consciousness raising, through which everybody encourages each other. However, intersectional conversations could also be seen as contextual, in the sense that, each member in the focus group could obtain more self-confidence as an individual in their working life by these reflective dialogues (Hill Collins, 1990/2000). It occurs though the participants drawing parallels and reflecting on their own and shared experiences, and by engaging in critical discussions in order to recreate one's own and other's (professional) situations. But, it also occurs by confirming each other's experiences, and by being open to acknowledging the other in the conversations.

The process then results in identification and an understanding that power relationships in the society are of central importance, and could be connected to the professional roles in the construction of the subjectivity (gender, ethnicity, etc.) at the workplace. In this case, it is important to note that the staff see their different ethnical backgrounds as being an invisible part of themselves, while, on the country, it is seen as very visible—and important—to their employers, as they intend to use this as a means of attracting older people from different backgrounds and therefore build the business.

Through an intersectional perspective, during the session, different tensions between staff members came out into the open, based on normative images of “power and subjectivity” (gender and ethnicity roles) that had not been questioned in the past. However, the working group is generally positive to the cooperation between different genders and ethnicities in the workplace, even if some are critical of what they called women's “harping on about things.” This was problematized by the researchers and explained as “subjective strategies.” That subjectivity (gender, ethnicity, etc.) created through an action can be explained with the aid of subordinate power constructions, which when they are made visible in a conversation can be converted into understanding and in that way grant experiences of “one's own power” (De los Reyes & Mulinari, 2005; Likke, 2007). The staff experiences and professional activities (in this study in the field of elderly care) can in that way be related to (power-) relational structures. Representations of subjectivity (gender, ethnicity, etc.) are not created in terms of general assumptions, but this act of assuming is constructed through power structures in society. Gender and ethnicity (subjectivity) can thus contribute to structural discrimination when this connection is not brought up in conversation (Acker, 2008).

Awareness of the intersectional perspective has helped the participants in the focus group to reflect on their knowledge sometimes being based on “intersectional structures.” Types of knowledge are contextually constructed and are not separate from the social tensions, practices, and power relationships that control the framework for how knowledge is produced (De los Reyes & Mulinari, 2005). It is apparent in the focus group that a “process of socialisation” is involved when the staff learn to take responsibility for the organization. The employer used this, since it disarmed the staff and gave them less room and opportunity to contemplate their own roles in relation to the power relationships within the organization.

Attaching an intersectional perspective opens up for “a questioning of old and new truths” (De los Reyes & Mulinari, 2005). It also promotes a more self-critical reflexive thinking in the focus group that affects their well-being. It is clear from the focus group that the staff develop feelings of guilt that they have difficulty processing, and that they relate this to their gender, that is, that women are expected to be caring individuals. When they cannot live up to their own demands feelings of vulnerability arise, due to the staff having an emotional connection to the older people in the care institution. They assume responsibility for taking care of them, despite not having the time. It is also apparent from the conversations in the focus group that the staff members of non-Swedish origin have felt singled out by their employer. They were asked to participate in the focus group based on the justification that they had a greater need than other staff to talk about their work. Obviously this statement created friction in the group.

The intersectional perspective also involved the role of the researcher. The research group represents different fields of knowledge and the individual researchers act differently based on their roles. One of the researchers had previous experience of working in care and participatory research in the area, which meant she could substantiate the experiences of the staff, and thus her pre-existing knowledge contributed to increased knowledge in this context, as (R1) actively participated in the conversations. At the same time, the other researcher (R2) was curious and could ask the staff to explain their situation, and then abstract this to a perspective connected to gender and ethnicity (subjectivity) in a more general context. Her pre-existing knowledge was based on gender studies and intersectionality, and she contributes to different explanatory models by asking questions in order to foster self-criticism in the participants. The researchers also complement each other as individuals. One has a non-Swedish ethnic background, which opened up for recognition in the working group, while the other one is of Swedish descent.

The staff experiences of professionalism and collaboration are constructed as a “dichotomy” of (power) structures on the one hand, and subjective interactions influenced by different cultural encounters on the other. The position for an individual in the collaborative work is influenced by this structure, which sometimes results in feelings of irritation towards each other. The intersectional dialogues made it clear that their working conditions is affected by power structures and power relations in the society on a structural level which reduced their feelings of guilt on a personal level. The majority of the staff was women and their different ethnicities are seen as an additional resource in their professional activities. Their professionalism is now permeated by an increased awareness of how power structures in society affect their perception of gender and ethnicity in relation to their everyday work.

## Conclusion

It is impossible to separate the global processes of the present day from the social dynamics of the workplace. This study shows that the work in elderly care is characterized by power structures in society and these affect positions in the workplace, affecting the employees’ well-being. The intersectional perspective contribution facilitates a better understanding of power structures through deeper reflections concerning the employees’ subjectivity (gender, ethnicity, etc.). In the course of the conversations connected to the focus group, the intersectional perspective was integrated in such a way that the experiences of the staff were legitimized while, at the same time, they acquired a more in-depth knowledge and understanding concerning their professional activities which contributed to their well-being.
